# Maternal knowledge, attitude, and perception about childhood routine immunization program in Atakumosa-west Local Government Area, Osun State, Southwestern Nigeria

**DOI:** 10.11604/pamj.supp.2021.40.1.30876

**Published:** 2021-11-12

**Authors:** Elizabeth B. Adedire, Olufemi Ajumobi, Omotayo Bolu, Patrick Nguku, Ikeoluwapo Ajayi

**Affiliations:** 1 African Field Epidemiology Network, Abuja, Nigeria,; 2 School of Community Health Sciences, University of Nevada, Reno, USA,; 3 Centers for Disease Control and Prevention, Atlanta, Georgia, USA,; 4 Epidemiology and Medical Statistics Department, College of Medicine, University of Ibadan, Oduduwa Road, 200132, Ibadan, Nigeria

**Keywords:** Child Immunization, mothers, knowledge, attitudes, health surveys, Nigeria

## Abstract

**Introduction:**

Routine Immunization (RI) is a key strategy in prevention of vaccine-preventable diseases (VPD). The Nigerian Demographic and Health survey 2013 showed that only 55% of children were fully immunized in Osun State. Historically, efforts to improve uptake of RI focused on health system factors with little attention on maternal related factors. This study assessed mothers´ knowledge, attitude, and perception towards the RI program in Atakumosa West Local Government Area (LGA) of Osun State.

**Methods:**

A total of 750 mothers were enrolled in a household survey using WHO cluster sampling in Atakumosa West LGA. Semi-structured questionnaires were used to obtain data on sociodemographic characteristics, knowledge on RI, attitudes, and perception of mothers towards RI program. Knowledge scores of ≥ 4 points based on six-point domain questions were regarded as good.

**Results:**

The mean (±SD) age of the mothers was 27.9 (± 6.1) years; 76% (571/750) had good knowledge of RI and VPD and a majority demonstrated a positive attitude towards the RI program. Antenatal care (ANC) attendance [OR 3.7; 95% CI (2.0 - 6.7)] health facility delivery [OR 1.7 (1.2 - 2.7)]; higher level of education [OR 1.9; 95% CI (1.4 - 2.5)], and mothers´ tetanus toxoid immunization status [OR 4.0 (2.3 - 7.2)] were significantly associated with having good knowledge of the RI program.

**Conclusion:**

A high proportion of mothers in Atakumosa West LGA of Osun State have good knowledge on childhood RI program. Current efforts at health education in ANC should be sustained and other strategies to improve knowledge on immunization need to be identified.

## Introduction

Immunization remains one of the most important public health interventions and a cost-effective strategy to reduce both the morbidity and mortality associated with vaccine-preventable diseases (VPDs). The World Health Organization (WHO) in 1974 initiated the Expanded Programme of Immunization (EPI), which aims to immunize, and thus protect, children against the following six deadly diseases: childhood tuberculosis, measles, poliomyelitis, diphtheria, pertussis, and tetanus [1]. Since that time, WHO added vaccination against hepatitis B and *Haemophilus influenza* type b to EPI, currently as pentavalent diphtheria-tetanus-pertussis-Hib-hep B vaccine.

In Nigeria, EPI was introduced in 1979, although RI is provided free of charge, VPDs remain the most common cause of childhood mortality with an estimated 40% death resulting from diseases that can be prevented by vaccination such as pneumonia, measles, and neonatal tetanus [2]. It is estimated that out of the six million children born every year in Nigeria, more than one million fail to complete appropriate vaccinations by their first birthday [3]. Despite a high level of political will demonstrated by government and appreciable contributions made by development partners to boost immunization service delivery, the proportion of children who received all doses of antigens in the EPI program across the states in Nigeria still ranged from 1.4% to 62.4% [4-7]. The Nigeria Demographic and Health Survey (NDHS) 2013 report indicated a slight decline in the proportion of fully-immunized children aged 12-23 months in Osun State from 57.8% in 2008 to 55.3% in 2013 [4]; the WHO target is 80% in all districts. Children in rural areas were 50% less likely to receive full vaccination than those in urban areas, thus contributing to the high morbidity and mortality due to VPDs among children in these areas [4].

The uptake of vaccination services is dependent not only on availability of and accessibility to vaccination services but also other factors including knowledge and attitude of mothers. Studies have shown that understanding the maternal perceptions and knowledge about immunization helps health planners develop effective health education programs and messages [8-10]. While the reasons for low immunization coverage have been proffered in general, mitigation efforts have focused on health system factors, but little attention has been paid to maternal knowledge, perception, beliefs, and practice. Understanding mothers´ knowledge and attitudes towards immunization could guide this aspect of multi-pronged efforts to improve routine immunization coverage.

This study is a part of a larger survey conducted in 2013 that assessed immunization coverage and determinants in Atakumosa-west Local Government Area (Nigeria district level), Osun State, Nigeria [11]. We assessed mothers´ knowledge, attitudes and perceptions on the routine childhood immunization program and examined associations with sociodemographic variables.

## Methods

### Study setting

Osun State is located in southwestern region of Nigeria with an estimated population in 2013 of 4,274,858 people, with 17% children under the age of five years [12]. Osun State has a total of 30 Local Government Areas most of which are predominantly rural. There are 736 public health facilities (678 primary, 54 secondary and 4 tertiary facilities) and 359 private health facilities. Over 88% of the public health facilities systematically offer routine immunization services [13].

### Study design

The cross-sectional study design included mothers of children aged 12-23 months who have been residing in the study area for at least 24 months. The survey sample size was determined using the methods recommended by the WHO for Immunization Coverage Cluster Survey (2005) for estimating a proportion. The sample size need was 750, assuming a proportion of fully immunized children = 58% [14], precision = ±5%, design effect = 2, and an alpha = 0.05. Atakumosa-west Local Government Area (LGA) was selected randomly from the 30 LGAs in Osun State. A two-stage sampling technique was then used to sample eligible mothers from the LGA. At stage one (selection of clusters), 30 clusters were selected from the available 170 clusters based on probability-proportional-to-size of the population. In stage two (selection of households), 25 households were selected from each of the 30 clusters selected at stage one. The first household in each cluster was selected randomly and subsequent households were selected contiguously on the right until the required number of eligible households for that cluster was achieved. From each selected household, one eligible mother was arbitrarily selected.

### Data collection and analysis

Data for the study were collected by 15 trained community health extension workers using a standardized structured and pretested interviewer-administered questionnaire. Data collected include sociodemographic characteristics of mothers and children, knowledge of mothers regarding routine immunization and VPDs; sources of information on the RI program; and the mother´s attitudes toward and perceptions of the program. Data analysis was performed using Microsoft Excel 2007 and SPSS Statistics for Windows, Version 19 (Armonk, NY: IBM Corp.) and analysis accounted for the cluster design using standard survey methods. Univariate data analyses were done to obtain frequencies, means and proportions. Bivariate data analyses were also carried out to identify factors associated with mothers having satisfactory knowledge of the childhood Immunization program.

### Study variables

To assess the knowledge of mothers on RI and VPDs, responses were scored using six questions on various aspects of routine immunization. The questions assessed respondent´s ability to state: 1) the correct purpose of immunization; 2) correct age a child should receive second doses of RI vaccinations; 3) age a child should receive the last doses of RI vaccines; 4) total number of visits a child should make to the health facility to receive all recommended doses; 5) at least three symptoms of VPDs; 6) at least three VPDs. Each correct response was scored one point while each wrong response was scored zero. Mothers who scored three points and below were graded as having poor knowledge while those who scored four points and above were graded as having good knowledge. This scoring system is similar to what has been used in a previous study in determining vaccination coverage in Nigeria [15]. To assess the attitudes of mothers, responses were graded on five attitude-related statements on RI using a Likert scale. The scores were then categorized into three groups: agree (strongly agree and agree), neutral (neither agree nor disagree) and disagree (disagree and strongly disagree).

### Ethical consideration

Ethical clearance for the study was obtained from the Ethical Review Committee of the Ministry of Health of Osun State. Written informed consent was obtained from each respondent and data were anonymized. Confidentiality of the respondents was assured and maintained during and after the study.

## Results

### Sociodemographic characteristics of the mothers

A total of 750 mothers were recruited for the study. The mean age of the mothers was 27.9 years (SD±6.1) and ranged 15-50 years. The majority (73.9%) were Christians and about half (55.3%) completed secondary school education; almost all were married (94.1%) and the highest proportion were traders (43.6%) (Table 1).

**Table 1:** sociodemographic characteristics of mothers in Osun State immunization knowledge survey, Nigeria, 2013

Characteristics	Frequency (n=750)	Proportion (%)
**Age-group (years)**		
<20	39	5.2
20-29	415	55.3
30-39	251	33.5
≥40	45	6.0
**Religion**		
Christian	554	73.9
Muslim	187	24.9
Traditional	9	1.2
**Education**		
No formal education	23	3.1
Completed primary	172	22.9
Completed secondary	402	55.3
Had some tertiary or more	153	20.4
**Marital Status**		
*Single	44	5.9
Married	706	94.1
**Mothers occupation**		
Trader	327	43.6
Artisans	157	20.9
Civil servant/formal employment	110	14.6
Farmer	92	12.3
Unemployed	64	8.6
**ANC attendance**		
Yes	703	93.7
No	47	6.3
**Heath facility delivery**		
Yes	611	81.5
No	139	18.5

* Single = widowed, separated, divorced and never married

### Knowledge of mothers on routine immunization and VPDs

**Figure 1:**
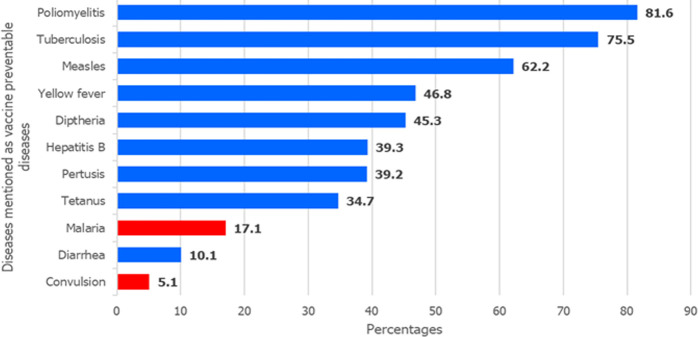
Knowledge of respondents on diseases preventable by vaccine

Overall, 77.9% of mothers mentioned the correct purpose of immunization, and 85.3%, 70.7% and 93.1% could correctly state the age at which each of the first, second and last doses of vaccine was given to children under one year, respectively. Overall a total of 571 mothers (76.1%) had good knowledge on VPDs and RI (Table 2). Most of the respondents correctly mentioned VPDs including poliomyelitis (81.6%), tuberculosis (75.5%), measles (62.2%) and yellow fever (46.8%). However, some mothers incorrectly mentioned malaria (17.1%), and convulsions (5.1%) as VPDs (Figure 1).

**Table 2:** knowledge of mothers on routine immunization and VPDs, Osun State immunization knowledge survey, Nigeria, 2013

Knowledge	Correct response (%)	Incorrect response (%)
Purpose of immunization (prevention of childhood diseases)	584 (77.9)	166 (22.1)
Age at which first dose is given (at birth)	617 (85.3)	133 (17.7)
Age at which second dose is given (6 weeks)	530 (70.7)	220 (29.3)
Age at which last dose is given (9 months)	698 (93.1)	52 (6.9)
Number of visits to health facility for RI (5 times)	608 (81.1)	142 (18.9)
Knowledge of at least 3 VPD	598 (79.7)	152 (20.3)
Knowledge of at least 3 symptoms of VPD	116 (15.5)	634 (84.5)

### Sources of information on routine immunization program

Overall, 71.9% mothers reported that they had access to RI information in the last twelve months and 498 (66.4%) had heard information on the benefits of routine immunization. The most common source of information reported by mothers was from health facilities; 199 (36.9%), radio programs 157 (29.1%); town criers 92 (17.0%) and television programs 35 (6.4%). Other mentioned sources of information on RI program included religious gatherings 38 (7.0%) and community meetings 14 (2.6%).

### Attitudes and perception of mothers towards routine immunization program

A total of 723 mothers (96.4%), stated that immunization is beneficial to children, and 98.5% agreed that childhood vaccines are safe. Most respondents (82.4%) would advise other mothers to take their children for routine immunization. Six hundred and ninety-four (92.6%) confirmed that RI services are provided free of charge. Very few mothers had contrary attitudes that immunization can cause infertility in life (0.9%), that government promotes immunization for its selfish interests (1.2%) and that there are local substitutes for routine immunization (6.4%) (Table 3).

**Table 3:** attitudes and perception of mothers towards routine immunization program, Osun State immunization knowledge survey, Nigeria, 2013

Attitude and perception statement	Agree	Disagree	Neither agree nor disagree
Immunization is beneficial	723 (96.4)	17 (2.3)	10 (1.3)
RI childhood vaccines are safe	739 (98.5)	2 (0.3)	9 (1.2)
All mothers should take their children for Immunization	618 (82.4)	132 (17.6)	0 (0.0)
Routine Immunizations are provided free of charge	694 (92.6)	28 (3.7)	28 (3.7)
Immunization can cause infertility later in life	7 (0.9)	701 (93.5)	42 (5.6)
Government promotes RI for selfish interest	9 (1.2)	669 (89.2)	72 (9.6)
Local preparation can serve as substitute for RI	48 (6.4)	630 (84.0)	72 (9.6)

### Association between sociodemographic factors and maternal knowledge on RI

Following a bivariate analysis, four factors were significantly associated with having good knowledge on childhood routine immunization program: 1) completing a higher or tertiary educational level (1.9 [1.4 - 2.5]); 2) receiving antenatal care (3.7 [2.0 - 6.7]; 3) delivery of their child at the health facility (1.7 [1.2 - 2.7]); 4) receiving tetanus immunization during pregnancy (4.0 [2.3 - 7.2] (Table 4).

**Table 4:** association between sociodemographic factors and maternal knowledge on routine immunization issues, Osun State immunization knowledge survey, Nigeria, 2013

Variables	Good knowledge n=571	Poor knowledge n= 179	Total N=750	OR [95% Confidence Interval]
**Age of mother (years)**				
≥30	216 (73.0)	80 (27.0)	296	0.8 [0.5 - 1.1]
<30	355 (78.2)	99 (21.8)	454	
**Marital status**				
Married	540 (76.5)	166 (23.5)	706	1.4 [0.7 - 2.7]
Single	31 (70.5)	13 (29.5)	44	
Education				
Secondary/higher	441 (79.5)	114 (20.5)	555	1.9 [1.4 - 2.5] **
No formal/primary	130 (66.7)	65 (33.3)	195	
**Monthly income**				
<N10000	312 (74.8)	105 (25.2)	417	0.9 [0.6 - 1.2]
≥N10000	259 (77.8)	74 (22.2)	333	
**ANC attendance**				
Yes	548 (78.0)	155 (22.0)	703	3.7 [2.0 - 6.7] **
No	23 (48.9)	24 (51.1)	47	
**Health facility delivery**				
Yes	478 (78.2)	133 (21.8)	611	1.7 [1.2 - 2.7] **
No	93 (66.9)	46 (33.1)	139	
**Mothers tetanus toxoid immunization**				
Yes	547 (78.3)	152 (21.7)	699	4.0 [2.3 - 7.2] **
No	24 (47.1)	27 (52.9)	51	

** Statistically significant, p<0.05 ***Mothers who scored three points and below were graded as having poor knowledge while those who scored four points and above were graded as having good knowledge

### Financial disclosure

The funding for this study was provided by USAID through a cooperative agreement between the Centers for Disease Control and Prevention, Atlanta and AFENET.

## Discussion

This study assessed mothers, knowledge, attitude, and perceptions regarding the routine immunization program for children in one LGA in Osun state. The knowledge of mothers on VPDs and routine immunization in this survey was satisfactory. Overall, more than 70 percent of mothers had good knowledge.

Our findings are similar to a 2006 survey in rural Edo State in southwestern Nigeria that showed 87% of respondents had satisfactory knowledge on routine immunization issues [15]. However, a similar survey conducted in 2010 in Zamfara State in northwest Nigeria, a state with much lower RI coverage in 2008 [14], revealed poor knowledge of mothers on routine immunization [16]. In our study, more than three-quarters of mothers knew the correct purpose of routine immunization, which is to prevent childhood illnesses. This finding is similar to a survey in another setting with higher RI coverage in Ethiopia, that revealed that ~80% of mothers mentioned that vaccination is to prevent childhood disease [17]. More than 80% of the mothers knew the appropriate schedule for immunization in terms of the number of visits to be made to the facilities in order to be fully immunized, the correct age at which the different vaccines are given particularly the correct age of the first and last doses. This is consistent with findings in southwestern Nigeria [15]. About 80% of mothers could mention three or more VPDs correctly, with high proportions readily identifying poliomyelitis, measles and tuberculosis as VPDs, similar to the findings in Zamfara [16]. Although mothers knew the correct routine immunization schedule and the purpose of the RI program, their knowledge on symptoms of individual VPDs was very low, contrary to the findings in Edo [15].

Even though this survey demonstrated positive perceptions of and attitudes about routine immunization. Certain misconceptions and negative beliefs still exist among a small proportion of the respondents. Contrary to findings from a 2010 qualitative study on immunization in Uganda [18], some mothers believe that children can still contract these VPDs whether they are immunized or not. This could potentially influence why some mothers do not take their children for any vaccination. Nonetheless, a much higher proportion would advise other women to take children for immunization as the benefits far outweighs the harm. These findings on providing advice to others are in accordance with a study reported in 2012 in Oyo State, also located in southwest Nigeria [19].

Our study shows that attendance of ANC and delivery at a health care facility was significantly associated with mothers´ knowledge on RI and VPDs; this corroborates with other studies conducted in the south of Nigeria [10,15]. It is important to note that 40% of responding mothers obtained information on the RI program primarily from the health facilities, which highlights the need to train and retrain health care workers on emphasizing the benefits of immunization and educating the mothers on VPDs, including during RI outreach sessions in the communities. One of the strengths of this survey is that it enrolled mothers within the communities thereby eliminating the selection bias that would have resulted if the respondent were drawn from mothers attending health facilities such as in some studies [20,21].

This report has limitations. One major limitation is that significant associations were found but given covariation of some of the factors; the means to address the issues affecting low knowledge and perceptions still require further exploration. The survey was limited to maternal knowledge and attitudes therefore did not explore paternal, family (e.g., other wives) and other potential sources of influencing a mother´s attitude towards routine immunization. These factors have been implicated by other studies in Tanzania and Iraq [22,23]. Likewise, our survey results cannot be considered representative of Osun State, since it only collected data from one of 30 LGAs. Lastly, the most recent Demographic and Health Survey in Nigeria was conducted in 2018 and reported in 2019. The results for receipt of all basic vaccination in Osun state in NDHS 2018 was 33.8%, a marked decline in the >50% full immunization found in the 2008 and 2012 NDHS results [24]. The knowledge and attitudes of women may have changed since that time in Atakumosa-west LGA.

## Conclusion

The findings of this study show overall satisfactory maternal knowledge and positive attitudes and perceptions regarding the childhood RI program. Attendance of antenatal care, health facility delivery of child and higher level of education were positively associated with having good knowledge of the immunization program and VPDs. Future efforts are needed to improve maternal knowledge and address misconceptions that may limit vaccination coverage rates in Osun and other regions of Nigeria and further issues explored on how to enhance knowledge and practices.

### Recommendation

Promotion of female education will help to reinforce knowledge of health issues critical to implementing child survival interventions. There is a need to reinforce health education on routine immunization among mothers during all health facility and RI outreach sessions. In addition, there is a need to explore the role of potential influencers inside and outside a household in the low uptake of routine immunization in Osun State.

### What is known about this topic

- Routine childhood immunization plays an important role in preventing childhood diseases;
- Mothers are the primary caregivers of their children and thus play an influential role in health status of their children; this has been elaborated in many studies;
- Studies have demonstrated that some sociodemographic factors affect knowledge, attitude, and perception of mothers with regard to their children´s immunization. Lack of comprehensive information and limited awareness about routine immunization results in poor uptake and utilization of RI services.

### What this study adds

- Maternal education is a strong predictor of good knowledge on immunization and vaccine preventable diseases in general and ultimately child immunization status;
- Mothers´ knowledge on immunization as well as utilization of routine immunization can be improved by addressing negative perception and misconception that may limit utilization of immunization services;
- This study demonstrates the necessity of enhancing technical capabilities and interpersonal communication skills of healthcare workers involved in maternal and child health services, as the most common source of information on routine immunization was from health facilities.
